# High-Dose Shilajit Enhances Xenograft-Mediated Bone Regeneration in a Rat Tibial Defect Model: An In Vivo Experimental Study

**DOI:** 10.3390/life15101528

**Published:** 2025-09-28

**Authors:** Ridvan Guler, Ersin Ozden, Firat Asır, Belgin Gulsun

**Affiliations:** 1 Department of Oral and Maxillofacial Surgery, Faculty of Dentistry, Dicle University, 21280 Diyarbakir, Turkey; bgulsun@gmail.com; 2 Department of Oral and Maxillofacial Surgery, Faculty of Dentistry, Firat University, 23119 Elazig, Turkey; ersinedzo@gmail.com; 3 Department of Histology and Embryology, Faculty of Medicine, Dicle University, 21280 Diyarbakir, Turkey; firatasir@gmail.com

**Keywords:** Shilajit, xenograft, bone regeneration, oxidative stress, TNF-α, histology, immunohistochemistry

## Abstract

Shilajit, a natural herbo-mineral compound with potent antioxidant, anti-inflammatory, and osteogenic properties, has been traditionally used to promote tissue repair. However, limited experimental data exist on its localized application in bone regeneration. This study aimed to evaluate the combined effect of Shilajit and bovine-derived xenograft on bone healing in a rat tibial defect model. Twenty-eight male Sprague–Dawley rats were randomly assigned to four groups (*n* = 7): Control (defect left to heal spontaneously), Graft-only, Graft + Shilajit 150 mg/kg, and Graft + Shilajit 250 mg/kg. Standardized 3 mm tibial defects were created and filled with xenograft in all groups except the Control. Shilajit was administered intraperitoneally on days 0–3 postoperatively. After 4 weeks, serum total oxidant status (TOS), total antioxidant status (TAS), and TNF-α levels were measured. Tibial specimens underwent histopathological, histomorphometric, and TNF-α immunohistochemical analysis. High-dose Shilajit significantly increased TAS and reduced TOS compared with the Control and Graft-only groups (*p* < 0.001). Median TNF-α concentrations decreased in a dose-dependent manner, with the lowest values in the high-dose group (15.7 [14.3–17.1] pg/mL, *p* < 0.001). Histomorphometry revealed the highest new bone area percentage (78.1% [74.9–81.2]) and lowest fibrous tissue content (9.8% [8.1–11.6]) in the high-dose group. Immunohistochemistry confirmed marked suppression of TNF-α expression in Shilajit-treated groups, particularly at high doses. The combination of Shilajit and bovine-derived xenograft significantly enhanced bone regeneration in a dose-dependent manner, likely through antioxidative, anti-inflammatory, and osteogenic mechanisms. These findings suggest that Shilajit may serve as a promising adjunct in bone grafting procedures.

## 1. Introduction

Bone regeneration is a major clinical challenge in oral and maxillofacial surgery, particularly in cases of congenital defects, trauma, or post-resection reconstruction [1,2]. Conventional bone grafting remains the gold standard for repairing such defects, with autologous bone offering excellent biocompatibility but limited by donor site morbidity and restricted availability [3,4]. Consequently, alternative graft materials—including allografts, xenografts, and synthetic substitutes—have been widely investigated to overcome these limitations [3,4,5]. Bovine-derived xenografts, in particular, are valued for their biocompatibility, osteoconductive properties, and mineral composition, which closely resembles that of human bone [6,7,8]. However, despite their structural benefits, xenografts lack intrinsic osteoinductive potential, making the adjunctive use of bioactive agents an attractive strategy to enhance regeneration.

Natural compounds with antioxidant and anti-inflammatory activities have gained attention as potential modulators of bone healing. Shilajit (mumie, mineral pitch) is a herbo-mineral exudate obtained from mountainous regions such as the Himalayas and Central Asia [9]. Its bioactive profile includes fulvic acid, dibenzo-α-pyrones (DBPs), chromoproteins, and essential minerals such as calcium, magnesium, and potassium [10,11]. Preclinical and clinical evidence indicates that Shilajit exerts antioxidant [12,13,14], anti-inflammatory [11,15], osteogenic [10,16,17], and immunomodulatory [11] effects, making it a promising candidate for bone tissue engineering applications. Mechanistically, Shilajit may promote osteoblast proliferation, collagen synthesis, and mineralization, while downregulating osteoclast activity and pro-inflammatory cytokines such as tumor necrosis factor-α (TNF-α) [10,16,18,19]. Given that oxidative stress and inflammation can impair bone repair and remodeling, strategies that improve redox balance and attenuate inflammatory signaling may support more effective regeneration around grafted sites [20,21].

Building on this rationale, the present study evaluated whether Shilajit used as a systemic adjunct can enhance xenograft-mediated bone healing in a standardized rat tibial defect model. We integrated biochemical (total antioxidant status [TAS], total oxidant status [TOS], and serum TNF-α), histological, histomorphometric, and immunohistochemical endpoints to provide a comprehensive assessment of regenerative outcomes. While prior work has explored Shilajit in bone-related contexts—including nanoparticle formulations and clinical settings [17,18,19,22]—there remains limited in vivo evidence on pairing Shilajit with xenograft scaffolds to support defect repair. We hypothesized that Shilajit, particularly at higher doses, would enhance bone regeneration through its antioxidative, anti-inflammatory, and osteogenic properties.

## 2. Materials and Methods

### 2.1. Preparation and Dosage of Shilajit

Shilajit, sourced from the Kyrgyzstan region, was washed, dried, ground to a fine powder, and dissolved in distilled water at a concentration reflecting traditional use. The solution was shaken for 24 h, centrifuged (5000× *g*, 10 min), sterilized by autoclaving, and reconstituted to the target doses. Two concentrations were prepared: 150 mg/kg and 250 mg/kg. The selected doses (150 mg/kg and 250 mg/kg) were based on previous animal studies investigating Shilajit’s pharmacological safety and efficacy. Prior toxicological assessments have reported no adverse effects in rodents at doses up to 500 mg/kg [15]. In addition, experimental studies evaluating Shilajit in bone and systemic models have demonstrated therapeutic efficacy at 150–250 mg/kg [18,23]. Accordingly, we chose 150 mg/kg as a low effective dose and 250 mg/kg as a higher therapeutic dose for comparison. Shilajit was administered intraperitoneally to the respective groups at 1 h, 1, 2, and 3 days after surgery.

### 2.2. Study Design

This in vivo experimental study was conducted at the Dicle University Experimental Research Center following approval from the Local Ethics Committee for Animal Experiments (Decision date: 30 October 2024; Decision no: 2024/43). All procedures complied with the Guide for the Care and Use of Laboratory Animals [24]. A total of 28 healthy male Sprague–Dawley rats (200–250 g) were housed under standard conditions (21 ± 1 °C, 40–60% humidity, 12 h light/dark cycle) with ad libitum access to food and water [24]. The minimum required sample size was determined by power analysis using G-Power software (version 3.1, Heinrich Heine University, Düsseldorf, Germany). Based on an effect size of 0.7, α = 0.05, and power = 0.80, the required sample size per group was calculated as 7 (Figure S1) [25]. Healthy male Sprague–Dawley rats (200–250 g) were included. Exclusion criteria were systemic illness, pre-existing injury, or development of postoperative complications requiring euthanasia (none occurred in this study).

Anesthesia was induced with intramuscular ketamine (50 mg/kg) and xylazine (5 mg/kg). After aseptic preparation, a 15 mm incision was made over the left tibial crest, and the cortical bone was exposed. A standardized defect (3 mm diameter, 4 mm depth) was created using a trephine drill under continuous saline irrigation [26]. In the graft-treated groups, the defect was filled with bovine-derived xenograft particles (particle size: 0.5–1 mm) (Botiss Biomaterials, Zossen, Germany). The flap was repositioned and sutured in layers with 4-0 polyglycolic acid. Postoperatively, all rats received intramuscular penicillin (50 mg/kg) and tramadol (0.1 mg/kg) for three days. Animals were monitored daily until euthanasia at week 4. The healing period was 4 weeks for all groups, after which animals were euthanized for biochemical, histological, and immunohistochemical analyses. All 28 animals completed the study period, and no deaths or unexpected complications occurred before the 4-week endpoint (Figure S2).

Control: Tibial defect left for spontaneous healing (no graft or Shilajit).

Graft-only: Defect filled with bovine-derived xenograft [26].

Graft + Shilajit 150 mg/kg: Xenograft plus intraperitoneal Shilajit (150 mg/kg) administered at 1 h, 1, 2, and 3 days post-surgery [18,23].

Graft + Shilajit 250 mg/kg: Xenograft plus intraperitoneal Shilajit (250 mg/kg) with the same dosing schedule [18,23].

### 2.3. Chemical Analysis of Shilajit Extract

Shilajit samples (Kyrgyzstan origin) were characterized for their phytochemical and elemental composition prior to experimental use. LC-MS/MS profiling, bioactive constituents, including fulvic acid, dibenzo-α-pyrones (DBPs), and related chromoproteins, were identified and quantified using an Agilent 6460 Triple Quad LC-MS/MS system [16,27]. Separation was achieved on a C18 reverse-phase column (150 × 4.6 mm, 5 µm) with a gradient of water (0.1% formic acid) and acetonitrile. Quantification was performed using calibration curves from authentic standards (R^2^ ≥ 0.99). Total phenolic content (TPC): Determined by Folin–Ciocalteu method, expressed as mg gallic acid equivalent (GAE) per g extract [16]. Antioxidant capacity: Evaluated using 2,2′-azino-bis-3-ethylbenzthiazoline-6-sulphonic acid (ABTS) and 1,1-diphenyl-2-picryl-hydrazyl (DPPH) radical scavenging assays, results expressed as IC_50_ values (µg/mL) [27].

### 2.4. Measurement of Serum TOS/TAS and TNF-α

At week 4, blood samples were collected from the abdominal aorta under anesthesia. Serum was separated by centrifugation (3000× *g*, 10 min, 4 °C) and stored at −80 °C until analysis [28]. Oxidative stress markers: Total oxidant status (TOS) and total antioxidant status (TAS) were measured using automated colorimetric assays (Rel Assay Diagnostics, Gaziantep, Turkey), following Erel’s method [28,29]. Results were expressed as µmol H_2_O_2_ equivalent/L (TOS) and mmol Trolox equivalent/L (TAS). Inflammatory marker: Serum TNF-α was determined using a rat-specific ELISA kit (Elabscience, Cat. No. E-EL-R0019, Houston, TX, USA) with a detection limit of 2.5 pg/mL. All samples were analyzed in duplicate, and mean values were used for statistical evaluation.

### 2.5. Histological and Immunohistochemical Analyses

Following euthanasia, left tibiae were harvested, fixed in 10% neutral-buffered formalin, decalcified in 10% Ethylenediaminetetraacetic acid (EDTA), dehydrated, and paraffin-embedded. Serial longitudinal sections (4 µm) through the defect center were stained with hematoxylin–eosin (H&E) for morphological assessment [30]. For histomorphometry, digital images (20×) were analyzed using QuPath v0.4.3. The defect area (DA) was delineated, and the new bone area (NBA, mm^2^) was measured as regions containing mineralized trabeculae with osteocytes. NBA% was calculated as (NBA/DA) × 100. Residual graft area (RGA%) and fibrous tissue area (FTA%) were quantified using color deconvolution and threshold segmentation [31]. Each sample was measured in triplicate, and mean values were used for analysis.

Immunohistochemistry for TNF-α (sc-52746, Santa Cruz Biotechnology, Dallas, TX, USA) was performed after deparaffinization, rehydration, antigen retrieval, peroxidase blocking, and incubation with primary antibody overnight at 4 °C. Detection was achieved with a biotin–streptavidin–peroxidase system and 3,3′-diaminobenzidine (DAB) chromogen, followed by hematoxylin counterstaining. Positive cell detection and DAB intensity quantification were performed in QuPath v0.4.3 using the H-DAB color deconvolution setting, avoiding artifacts or necrotic regions. Results were expressed as the percentage of TNF-α–positive cells. All histological and immunohistochemical evaluations were conducted by a blinded pathologist [32,33].

### 2.6. Statistical Analysis

Data analysis was performed using GraphPad Prism version 9.0 (GraphPad Software, San Diego, CA, USA; www.graphpad.com). The software was used to conduct normality testing (Shapiro–Wilk test), parametric analyses (one-way ANOVA with Tukey’s post hoc test), and non-parametric analyses (Kruskal–Wallis test with Dunn’s post hoc comparisons). Normally distributed variables were expressed as mean ± standard deviation (SD), while non-normally distributed variables were presented as median [interquartile range]. The relationship between new bone area percentage (NBA%) and serum TNF-α concentrations was evaluated using simple linear regression analysis and correlation analysis. The coefficient of determination (R^2^) was used to quantify the proportion of variance in TNF-α explained by NBA%, and the corresponding *p*-value tested the null hypothesis that the slope of the regression line equals zero. Regression analysis was performed for the pooled dataset (all animals combined) and visualized as a scatter plot with individual data points color-coded by group. A *p*-value < 0.05 was considered statistically significant for all tests. The software is widely used for statistical analysis and data visualization in biomedical sciences (Motulsky H. Prism 9 Statistics Guide. GraphPad Software, San Diego, CA, USA, Available at: https://www.graphpad.com/guides/prism/latest/statistics/index.htm, accessed on: 13 June 2025).

## 3. Results

### 3.1. LC–MS/MS Analysis Reveals Potent Antioxidant Activity of Shilajit Extract

LC-MS/MS analysis confirmed that the Shilajit extract contained high levels of fulvic acid (215.4 ± 4.8 mg/g) and measurable dibenzo-α-pyrones (12.7 ± 0.9 mg/g), consistent with previously reported bioactive profiles. The total phenolic content was 35.6 ± 1.1 mg GAE/g, and antioxidant assays demonstrated potent radical scavenging activity (ABTS IC_50_ = 48.2 µg/mL; DPPH IC_50_ = 62.5 µg/mL) (Table 1).

### 3.2. High-Dose Shilajit Enhances Antioxidant Capacity and Suppresses Oxidative and Inflammatory Markers

According to the results of serum biochemical analysis, the control group exhibited the lowest TAS (0.53 ± 0.09 mmol Trolox equiv/L) and the highest TOS (68.8 ± 1.48 µmol H_2_O_2_ equiv/L), indicating significant oxidative stress during the spontaneous healing process. Graft application led to a notable increase in TAS (0.95 ± 0.13 mmol/L, *p* < 0.05 vs. Control) and reduction in TOS (50.2 ± 4.69 µmol/L, *p* < 0.05 vs. Control). In the group receiving 150 mg/kg Shilajit, antioxidant capacity increased further (TAS: 1.09 ± 0.23 mmol/L, *p* < 0.05 vs. Graft-only) and oxidant levels decreased more markedly (TOS: 38.6 ± 1.61 µmol/L, *p* < 0.05 vs. Graft-only). The most significant changes occurred in the 250 mg/kg Shilajit group, where TAS reached the highest value (1.34 ± 0.27 mmol/L, *p* < 0.001 vs. Control and Graft-only) and TOS dropped to the lowest level (20.7 ± 3.01 µmol/L, *p* < 0.001 vs. all other groups), indicating effective suppression of oxidative stress and enhancement of systemic antioxidant defenses. These findings suggest that Shilajit modulates oxidative balance in a dose-dependent manner, which may contribute to its positive effects on bone healing (Table 2).

At week 4, serum TNF-α concentrations were significantly lower in all treated groups compared to the control group (*p* < 0.001) (Table 2). Median TNF-α levels decreased from 42.5 (40.1–45.0) pg/mL in controls to 31.4 (29.5–33.2) pg/mL in the graft-only group (*p* < 0.05 vs. Control). Shilajit supplementation further reduced TNF-α levels in a dose-dependent manner, reaching 22.8 (21.1–24.2) pg/mL in the 150 mg/kg group and 15.7 (14.3–17.1) pg/mL in the 250 mg/kg group (*p* < 0.05 for both vs. Graft-only). These results indicate that Shilajit supplementation, particularly at higher doses, exerts a pronounced dose-dependent anti-inflammatory effect by significantly suppressing systemic and local TNF-α levels, thereby creating a more favorable environment for bone regeneration.

### 3.3. High-Dose Shilajit with Grafting Maximizes Bone Regeneration and Histological Quality

**Figure 1:**
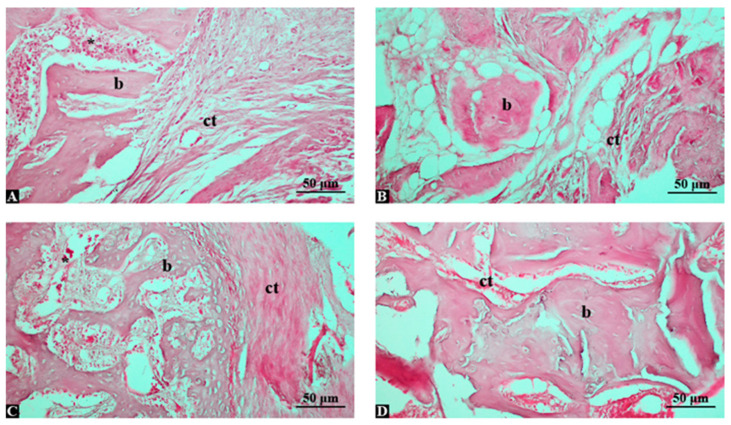
Hematoxylin and eosin-stained histological sections of tibial bone defect areas in different experimental groups (magnification: ×200; scale bar: 50 μm). (**A**) Control group: Limited new bone formation (b) and predominant fibrous connective tissue (ct) were observed within the defect area. (**B**) Graft-only group: Irregular new bone trabeculae (b) were noted around the graft material, accompanied by adipose tissue and loose connective tissue (ct). (**C**) Graft + low-dose Shilajit group (150 mg/kg): Moderately organized new bone trabeculae (b) were detected with reduced connective tissue (ct), indicating enhanced osteoblastic activity. (**D**) Graft + high-dose Shilajit group (250 mg/kg): Extensive and well-organized bone trabeculae (b) filled the defect area, with minimal fibrous tissue, reflecting marked bone regeneration. Abbreviations: b, bone tissue; ct, connective tissue, *: inflammation.

Since the control group was left to heal spontaneously, new bone formation was quite limited, and the defect area was mostly filled with fibrous connective tissue. The presence of inflammation was observed. Osteoblastic activity was low, while osteoclastic activity was prominent. These findings indicate the insufficiency of the natural healing process (Figure 1A). In the group treated with graft alone, new bone formation had initiated, and increased osteoblastic activity was observed around the graft particles. However, the graft had not yet transformed into a fully organized bone structure, and the presence of adipose tissue areas was notable. Although this reflects the osteoconductive effect of the graft, it also indicates that the regenerative process remains limited (Figure 1B). The graft application supported with low-dose Shilajit appears to enhance new bone formation. Bone trabeculae were more regularly aligned, osteoblastic activity increased, connective tissue formation decreased, and inflammation was less pronounced. These findings suggest that the anti-inflammatory and regenerative effects of Shilajit have begun to take place (Figure 1C). In the group treated with graft combined with high-dose Shilajit, the most intense new bone formation was observed. The trabecular structure was well-organized, and the bone tissue appeared close to a mature state. Osteoblastic activity was at its highest level, osteoclastic activity was reduced, and inflammation was minimal. These findings strongly support the dose-dependent bone-healing potential of Shilajit (Figure 1D).

### 3.4. Shilajit Extract Increases New Bone Area and Reduces Fibrous Tissue in a Dose-Dependent Manner

At 4 weeks, the median NBA% was lowest in the Control group (13.5 [11.9–15.2]) and significantly higher in the Graft-only group (33.6 [30.4–36.9], *p* < 0.05 vs. Control). Both Shilajit-supplemented groups showed marked improvements, with NBA% reaching 59.1 [55.8–62.7] in the Graft + 150 mg/kg group and 78.1 [74.9–81.2] in the Graft + 250 mg/kg group (*p* < 0.001 vs. Control and Graft-only). Residual graft percentage decreased in a dose-dependent manner with Shilajit administration (*p* < 0.05), while fibrous tissue content was lowest in the Graft + 250 mg/kg group (9.8 [8.1–11.6]) compared to all other groups (*p* < 0.001). Defect area measurements did not differ significantly between groups, confirming consistency in the initial defect size (Table 3).

### 3.5. High-Dose Shilajit Markedly Reduces Local TNF-α Expression During Bone Healing

**Figure 2:**
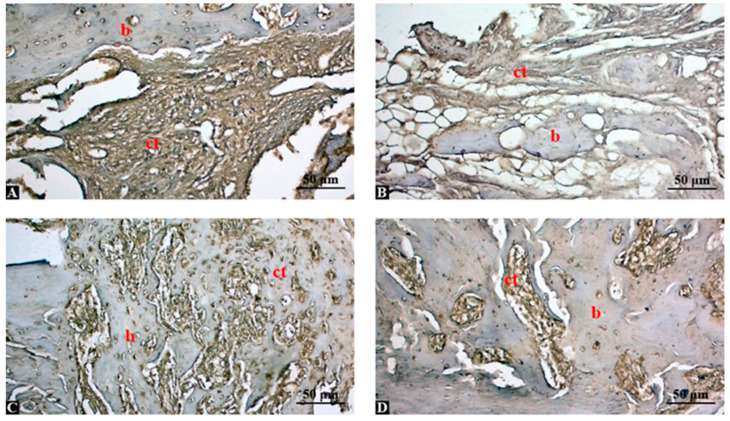
TNF-α immunohistochemical staining in tibial bone defect areas across experimental groups (magnification: ×200; scale bar: 50 μm). (**A**) Control group: Strong TNF-α immunopositivity in fibrous connective tissue (ct) and around bone (b), indicating high inflammatory activity. (**B**) Graft group: Moderate TNF-α expression in connective tissue (ct) and weak staining in bone (b), reflecting partial inflammatory suppression. (**C**) Graft + low-dose Shilajit (150 mg/kg): Decreased TNF-α immunoreactivity, with mild staining in connective tissue (ct) and limited positivity in bone (b). (**D**) Graft + high-dose Shilajit (250 mg/kg): Minimal TNF-α expression, particularly in connective tissue (ct), with nearly negative staining in bone (b), indicating effective inflammation reduction. Abbreviations: b, bone tissue; ct, connective tissue.

Bone sections were immune stained with TNF-α antibody and shown in Figure 2. In the control group, strong TNF-α immunopositivity was observed in fibrous connective tissue (ct) areas and around the bone surface (b), indicating ongoing inflammation and insufficient bone regeneration (Figure 2A). In the graft group, moderate TNF-α expression was detected, especially in connective tissue (ct) surrounding the graft particles. Bone tissue (b) showed weak immunoreactivity, suggesting partial inflammatory activity (Figure 2B). In graft + low-dose Shilajit group (150 mg/kg), TNF-α expression was reduced compared to the control and graft-only groups. Immunoreactivity was mainly localized to connective tissue (ct), while newly formed bone (b) areas showed mild staining, indicating decreased inflammation (Figure 2C). In the graft + high-dose Shilajit group (250 mg/kg), minimal TNF-α expression was observed, especially in the connective tissue (ct), and the bone (b) areas were largely immunonegative (Figure 2D). This suggests a significant anti-inflammatory effect and improved healing response.

### 3.6. Semi-Quantitative Analysis Confirms Dose-Dependent Suppression of TNF-α by Shilajit

Semi-quantitative analysis of TNF-α immune stainings is shown in Table 4. The control group exhibited the highest TNF-α expression, reflecting a strong inflammatory response in the absence of intervention. Graft application alone significantly reduced TNF-α levels, while the addition of low-dose Shilajit further suppressed inflammation. The most notable decrease in TNF-α expression was observed in the high-dose Shilajit group, supporting its potent anti-inflammatory effect during bone healing (*p* < 0.001).

### 3.7. Inverse Association Between New Bone Formation and Serum TNF-α Levels Across Experimental Groups

**Figure 3:**
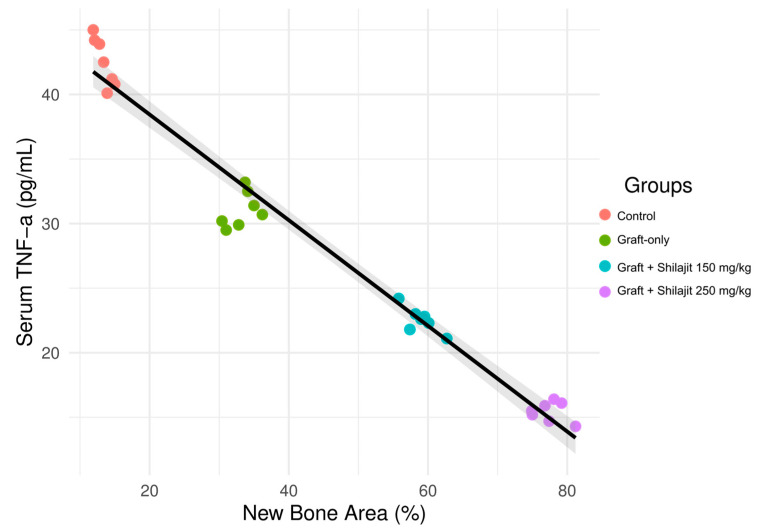
Scatter plot illustrating the pooled regression analysis between new bone area percentage (NBA%) and serum TNF-α concentrations at 4 weeks across all experimental groups (Control, Graft-only, Graft + Shilajit 150 mg/kg, and Graft + Shilajit 250 mg/kg). A single regression line with 95% confidence intervals is shown, demonstrating a significant inverse association between NBA% and TNF-α levels (R^2^ = 0.972, *p* < 0.001). Grey shaded area: Confidence interval.

The pooled regression analysis demonstrated a significant inverse association between new bone area percentage (NBA%) and serum TNF-α concentrations across all experimental groups. A negative significant correlation was determined between TNF-α levels and new bone area (NBA) (r_s_ = −0.95, *p* < 0.001). This indicates that an increase in TNF-α levels significantly reduces new bone formation (Figure 3). Higher NBA% values were consistently associated with lower TNF-α levels, and this relationship remained robust after pooling the data rather than fitting separate regression lines for each group. The addition of 95% confidence intervals provided a more conservative estimate of the correlation and highlighted the uncertainty of the regression fit.

## 4. Discussion

The present study demonstrated that the combination of Shilajit and bovine-derived xenografts significantly enhanced bone regeneration in a rat tibial defect model in a dose-dependent manner. High-dose Shilajit yielded the highest new bone area percentage, the most organized trabecular structure, and lowest inflammatory activity as indicated by TNF-α expression. These findings suggest that Shilajit, when used as an adjunct to bone grafting, provides synergistic benefits through its antioxidative, anti-inflammatory, and osteogenic properties.

Oxidative stress is known to impair bone healing by promoting osteoclastogenesis and reducing osteoblast function [20]. In our study, high-dose Shilajit significantly increased TAS and reduced TOS levels compared to both the control and graft-only groups, indicating improved systemic redox balance. This is consistent with previous reports showing that fulvic acid and dibenzo-α-pyrones in Shilajit act as potent free radical scavengers and enhance mitochondrial function [14,15,16,34]. By protecting bone-forming cells from oxidative damage, Shilajit likely creates a more favorable environment for mineralized tissue deposition.

Histomorphometric analysis confirmed that grafting alone improved bone fill compared to spontaneous healing, but the addition of Shilajit—particularly at high doses—resulted in a nearly two-fold increase in NBA% compared with the graft-only group. Residual graft area decreased, and fibrous tissue content was lowest in the high-dose group, indicating accelerated graft resorption and replacement by new bone. These observations support previous findings that Shilajit promotes osteoblast proliferation and collagen synthesis while suppressing osteoclast activity [12,18,35].

Inflammation is another critical modulator of bone repair. Elevated TNF-α can prolong the inflammatory phase and impair osteogenesis [21]. In our study, both serum TNF-α and local immunopositivity decreased in Shilajit-treated groups in a dose-dependent manner, with minimal expression in the high-dose group. This aligns with prior evidence that Shilajit downregulates pro-inflammatory cytokines and protects against tissue injury [13,17,23]. The negative correlation between TNF-α levels and NBA% further underscores the role of inflammation control in successful bone regeneration.

Our findings are in line with recent experimental and clinical evidence supporting Shilajit’s osteogenic potential. In a rat tibial defect model, Kangari et al. [12] reported that Shilajit enhanced the osteogenic differentiation of adipose-derived mesenchymal stem cells, resulting in increased osteoblast numbers, collagen deposition, and improved biomechanical strength. Similarly, a rabbit tibial defect study using Shilajit-stabilized silver nanoparticles demonstrated faster radiographic union and earlier mature bone formation compared to controls [22]. Beyond defect models, Shilajit has shown protective effects against bone loss in systemic conditions: in a glucocorticoid-induced osteoporosis model, Alshubaily and Jambi [18] found that Shilajit, particularly in nanoparticle form, significantly improved bone mineral content and architecture while reducing malondialdehyde and hydrogen peroxide levels and increasing antioxidant defenses such as total antioxidant capacity and superoxide dismutase. Clinical data also support its fracture-healing potential; in a randomized placebo-controlled trial, Sadeghi et al. [17] demonstrated that oral Shilajit shortened tibial fracture healing time without increasing adverse effects. Furthermore, in a year-long trial on postmenopausal women with osteopenia, Pingali and Nutalapati [19] showed that Shilajit preserved bone mineral density and favorably modulated bone turnover markers (decreased CTX-1 and RANKL, increased OPG), alongside reductions in oxidative stress and inflammatory markers. Collectively, these findings reinforce that Shilajit exerts synergistic effects with graft materials by providing both a mineral-rich scaffold and potent antioxidant and anti-inflammatory support, ultimately creating a microenvironment conducive to bone regeneration.

High-dose Shilajit may accelerate bone regeneration through a multifaceted mechanism. In addition to its antioxidant role, it could provide essential minerals and organic substrates for bone synthesis, enhance collagen matrix deposition, stimulate osteogenic cell differentiation and proliferation, suppress osteoclast activity, and modulate inflammatory signaling [17,18,19]. Recent in vivo studies in fracture and defect models, as well as in vitro experiments with mesenchymal stem cells and osteoblasts, support these potential effects [12,22,23]. The markedly higher NBA% observed at 250 mg/kg may therefore reflect the convergence of these complementary pathways, suggesting that a higher dose might reach a threshold where osteogenic, anti-resorptive, and immunomodulatory effects act synergistically to enhance bone fill.

This study has some limitations. The use of a single animal model and defect site may not fully replicate the healing dynamics of craniofacial bones. Additionally, the observation period was limited to four weeks, and long-term outcomes such as remodeling and functional integration remain unknown. Another limitation is the absence of a Shilajit-only (no graft) group. While our focus was on assessing Shilajit as an adjunct to xenograft application, such a group would help clarify whether Shilajit alone can induce bone regeneration. It should also be noted that the correlation analysis between NBA% and TNF-α yielded a very high coefficient of determination (R^2^ = 0.972). Given the relatively small sample size (*n* = 7 per group), such a near-perfect correlation carries a risk of overfitting and should be interpreted as exploratory rather than definitive. To address this concern, we re-analyzed the data using a pooled regression model with 95% confidence intervals instead of separate group-wise regression lines. This approach provides a more realistic estimate of the overall relationship and conveys the uncertainty of the regression fit. Finally, the molecular mechanisms underlying Shilajit osteogenic effects were not explored in detail and should be addressed in future studies. In addition, potential methodological bias cannot be completely excluded, as the study was performed in a single center using a single animal model and treatment schedule. Expanding research to include various graft types, extended healing durations, mechanistic analyses, and multicenter designs will help validate and extend the clinical relevance of these findings while minimizing potential methodological bias.

## 5. Conclusions

This experimental study demonstrated that Shilajit, particularly at a high dose, significantly enhances bone regeneration when used in combination with bovine-derived xenografts in a rat tibial defect model. The observed improvements—higher new bone area percentage, better trabecular organization, reduced fibrous tissue, and suppressed TNF-α expression—were accompanied by favorable shifts in systemic oxidative balance, with increased TAS and reduced TOS levels. These effects align with recent in vivo and clinical evidence indicating that Shilajit promotes osteogenesis through its potent antioxidant, anti-inflammatory, and mineral-rich composition. By synergizing with the osteoconductive framework of xenografts, Shilajit may create a regenerative microenvironment that accelerates defect healing and improves tissue quality. These findings support the potential clinical application of Shilajit. Future studies should focus on long-term outcomes, diverse bone sites, and mechanistic analyses to validate and expand these results for translational use.

## Figures and Tables

**Table 1:** Phytochemical and antioxidant profile of the Shilajit extract.

Parameter	Result	Unit/Expression
Fulvic acid	215.4 ± 4.8	mg/g extract
Dibenzo-α-pyrones	12.7 ± 0.9	mg/g extract
Total phenolic content	35.6 ± 1.1	mg GAE/g extract
ABTS radical scavenging	48.2 ± 1.6	IC_50_ µg/mL
DPPH radical scavenging	62.5 ± 2.0	IC_50_ µg/mL

ABTS: 2,2′-azino-bis-3-ethylbenzthiazoline-6-sulphonic acid; DPPH: 1,1-Diphenyl-2-picryl-hydrazyl.

**Table 2:** Serum TNF-α concentrations at 4 weeks.

Group (*n* = 7)	TAS (mmol Trolox equiv/L)	TOS (µmol H_2_O_2_ equiv/L)	TNF-α (pg/mL)
Control	0.53 ± 0.09	68.8 ± 1.48	42.5 (40.1–45.0)
Graft-only	0.95 ± 0.13 *	50.2 ± 4.69 *	31.4 (29.5–33.2) *
Graft + Shilajit 150 mg/kg	1.09 ± 0.23 **	38.6 ± 1.61 **	22.8 (21.1–24.2) **
Graft + Shilajit 250 mg/kg	1.34 ± 0.27 ***	20.7 ± 3.01 ***	15.7 (14.3–17.1) ***

TAS/TOS: (Total antioxidant status/Total oxidant status); One-way ANOVA (post hoc Tukey’s multiple comparison); TNF-α: Kruskal–Wallis (post hoc Dunn’s multiple comparison). Values are mean ± SD and median (IQR). * vs. control, ** vs. Graft only, *** vs. graft only and graft + Shilajit 150 mg/kg.

**Table 3:** Histomorphometric measurements of tibial defects at 4 weeks.

Group (*n* = 7)	DA (mm^2^)	NBA (mm^2^)	NBA%	RGA%
Control	7.05 (6.90–7.18)	0.95 (0.82–1.08)	13.5 (11.9–15.2)	0.0
Graft-only	7.01 (6.88–7.12)	2.35 (2.10–2.58)	33.6 (30.4–36.9)	28.4 (25.1–31.6)
Graft + Shilajit 150 mg/kg	7.03 (6.92–7.16)	4.15 (3.88–4.41)	59.1 (55.8–62.7)	17.6 (15.2–20.1)
Graft + Shilajit 250 mg/kg	7.02 (6.89–7.15)	5.48 (5.25–5.72)	78.1 (74.9–81.2)	8.9 (7.1–10.6)

DA: Defect Area, NBA: New Bone Area, RGA: Residual Graft Area. Values are median (IQR). Kruskal–Wallis (Dunn’s test, post hoc).

**Table 4:** Semi-quantitative analysis of TNF-α immune stainings is shown in Table 1.

Group	Positive Cell Rate (%)	Mean ± SD	Statistical Comparison
Control	68.2%, 70.4%, 66.9%, 69.1%, 67.5%, 71.0%, 68.8%	68.8 ± 1.5	
Graft-only	52.0%, 50.6%, 48.9%, 51.3%, 53.1%, 49.7%, 50.5%	50.9 ± 1.5	*p* < 0.0001 (vs. control)
Graft + Shilajit 150 mg/kg	38.7%, 36.9%, 35.2%, 37.8%, 36.5%, 38.3%, 37.0%	37.2 ± 1.1	*p* < 0.0001 (vs. graft-only)
Graft + Shilajit 250 mg/kg	22.1%, 23.8%, 21.4%, 22.9%, 23.5%, 20.7%, 22.6%	22.4 ± 1.0	*p* < 0.0001 (vs. graft-only and 150 mg/kg shilajit)

One-way ANOVA (post hoc Tukey’s multiple comparison).

## Data Availability

The original contributions presented in this study are included in the article/Supplementary Materials. Further inquiries can be directed to the corresponding author.

## Supplementary Materials

The following supporting information can be downloaded at: https://www.mdpi.com/article/10.3390/life15101528/s1, Figure S1: XY plot for G-power analysis; Figure S2: Surgical procedure of tibial defect creation and grafting. (A) Crestal incision along the tibial surface followed by careful elevation of the soft tissue to expose the cortical bone. (B) Standardized circular defect (3 mm diameter, 4 mm depth) created with a trephine drill under constant saline irrigation. (C) Placement of bovine-derived xenograft particles (0.5–1 mm) into the prepared defect site. No graft material was applied in the control group.

## Abbreviations

The following abbreviations are used in this manuscript:

TOS	Total oxidant status
TAS	Total antioxidant status
TNF	Tumor necrosis factor
H-E	Hematoxylin and eosin
DAB	3,3′-diaminobenzidine
DPB	Dibenzo-α-pyrones
TPC	Total phenolic content
GAE	Gallic acid equivalent
ABTS	2,2′-azino-bis-3-ethylbenzthiazoline-6-sulphonic acid
DPPH	1,1-Diphenyl-2-picryl-hydrazyl
DA	Defect area
NBA	New bone area
RGA	Residual graft area
FTA	Fibrous tissue area
SD	Standart Deviation
EDTA	Ethylenediaminetetraacetic acid
