# Supplementary material for: High-Dose Shilajit Enhances Xenograft-Mediated Bone Regeneration in a Rat Tibial Defect Model: An In Vivo Experimental Study
Source: Life (Basel). 2025 Sep 28;15(10):1528. doi: 10.3390/life15101528 (PMC12565024; doi:10.3390/life15101528)
Supplement: Supplementary file 1 [file life-15-01528-s001.zip › life-3853939-supplementary.pdf]

Supplementary Figures

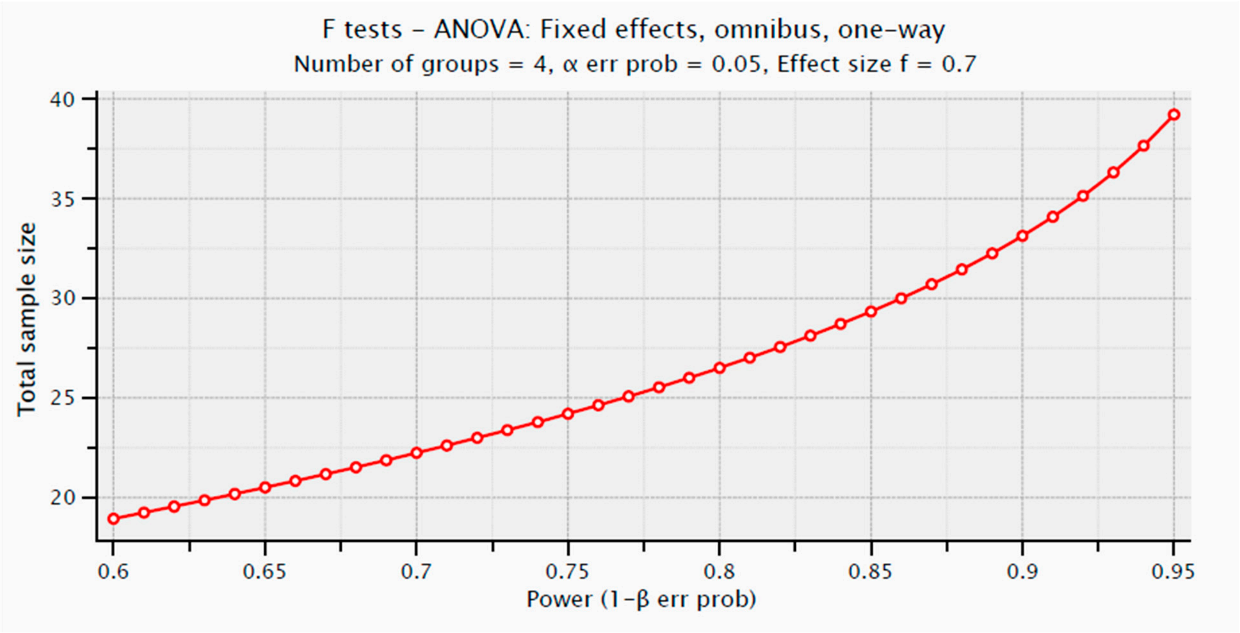

Figure S1. XY plot for G-power analysis.

Power Analysis

An a priori power analysis was conducted using G*Power software (version 3.1.9.7; Heinrich-Heine-Universität Düsseldorf). The test family was set to F tests, and the statistical test specified as ANOVA: Fixed effects, omnibus, one way. The type of power analysis was A priori: compute required sample size—given α, power, and effect size. Input parameters were: effect size f = 0.7, α = 0.05, power (1–β) = 0.80, and number of groups = 4. Based on these settings, the required total sample size was calculated as 28 animals (n = 7 per group).

Test results

F tests - ANOVA: Fixed effects, omnibus, one-way

Analysis: A priori: Compute required sample size

Input: Effect size f = 0.7

α err prob = 0.05

Power (1-β err prob) = 0.80

Number of groups = 4

Output: Noncentrality parameter λ = 13.7200000

Critical F = 3.0087866

Numerator df = 3

Denominator df = 24

Total sample size = 28

Actual power = 0.8279168

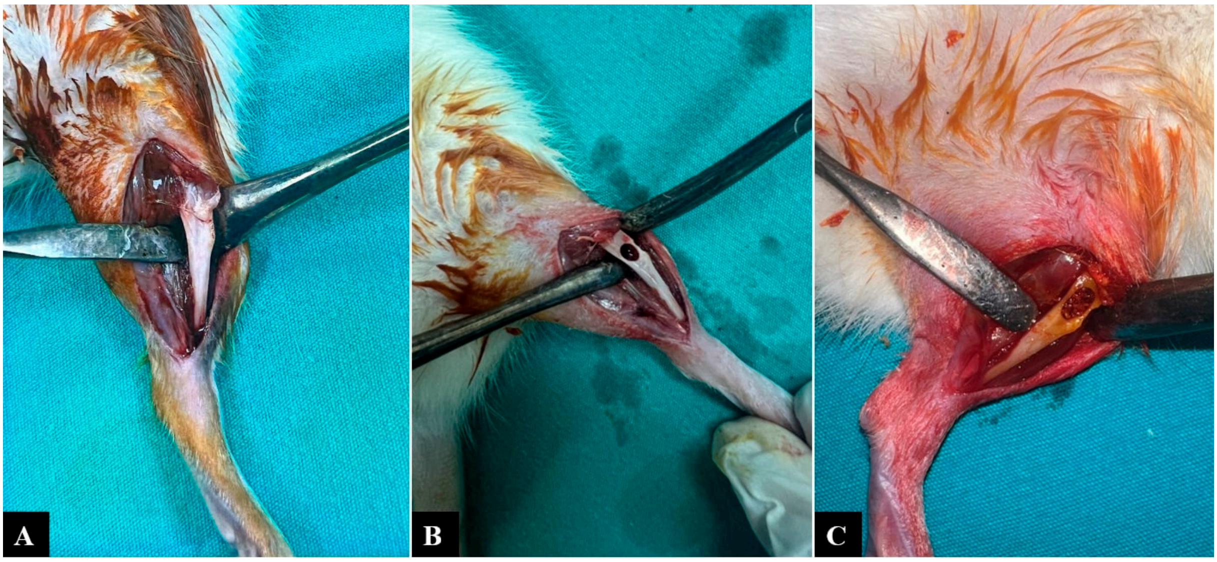

Figure S2. Surgical procedure of tibial defect creation and grafting. (A) Crestal incision along the tibial surface followed by careful elevation of the soft tissue to expose the cortical bone. (B) Standardized circular defect (3 mm diameter, 4 mm depth) created with a trephine drill under constant saline irrigation. (C) Placement of bovine-derived xenograft particles (0.5–1 mm) into the prepared defect site. No graft material was applied in the control group.
